# Uncovering the Metabolic Footprint of New Psychoactive Substances by Metabolomics: A Systematic Review

**DOI:** 10.3390/molecules30020290

**Published:** 2025-01-13

**Authors:** Ana Sofia Almeida, Paula Guedes de Pinho, Fernando Remião, Carla Fernandes

**Affiliations:** 1 Laboratório de Química Orgânica e Farmacêutica, Departamento de Ciências Químicas, Faculdade de Farmácia, Universidade do Porto, Rua Jorge Viterbo Ferreira, 228, 4050-313 Porto, Portugal; anasofiaalmeida1998@gmail.com; 2 Centro Interdisciplinar de Investigação Marinha e Ambiental (CIIMAR), Universidade do Porto, Terminal de Cruzeiros do Porto de Leixões, Avenida General Norton de Matos, s/n, 4450-208 Matosinhos, Portugal; 3 UCIBIO-Applied Molecular Biosciences Unit, Laboratory of Toxicology, Department of Biological Sciences, Faculty of Pharmacy, Universidade do Porto, Rua Jorge Viterbo Ferreira, 228, 4050-313 Porto, Portugal; pguedes@ff.up.pt (P.G.d.P.); remiao@ff.up.pt (F.R.); 4 Associate Laboratory i4HB—Institute for Health and Bioeconomy, Universidade do Porto, Rua Jorge Viterbo Ferreira, 228, 4050-313 Porto, Portugal

**Keywords:** metabolomics, new psychoactive substances, synthetic cannabinoids, synthetic cathinones, toxicity

## Abstract

New psychoactive substances (NPSs) emerged in the 2000s as legal alternatives to illicit drugs and quickly became a huge public health threat due to their easy accessibility online, limited information, and misleading labels. Synthetic cannabinoids and synthetic cathinones are the most reported groups of NPSs. Despite NPSs being widely studied, due to their structural diversity and the constant emergence of novel compounds with unknown properties, the development of new techniques is required to clarify their mode of action and evaluate their toxicological effects. Metabolomics has been a useful tool to evaluate the metabolic effects of several xenobiotics. Herein, a systematic review was performed, following PRISMA guidelines, regarding metabolomic studies on synthetic cathinones and synthetic cannabinoids to evaluate their effects in cellular metabolism. In the studies, *in vivo* models were the most employed (86%) and the analysis mostly followed untargeted approaches (75%) using LC-MS techniques (67%). Both groups of NPSs seem to primarily interfere with energy metabolism-related pathways. Even though this type of study is still limited, metabolomics holds great promise as a tool to clarify mechanisms of actions, identify biomarkers of exposure, and explain the toxicological effects of NPSs.

## 1. Introduction

New psychoactive substances (NPSs) emerged in the 2000s as legal alternatives to illicit drugs, being known as “legal highs”, “smart drugs”, or “research chemicals” [1]. These substances rapidly became a significant public health concern since they are easily accessible online, information regarding their pharmacological and toxicological properties is scarce, and often there are discrepancies between the labeled composition and the real content, leading to the consumption of unknown substances by users [2,3].

**Figure 1:**
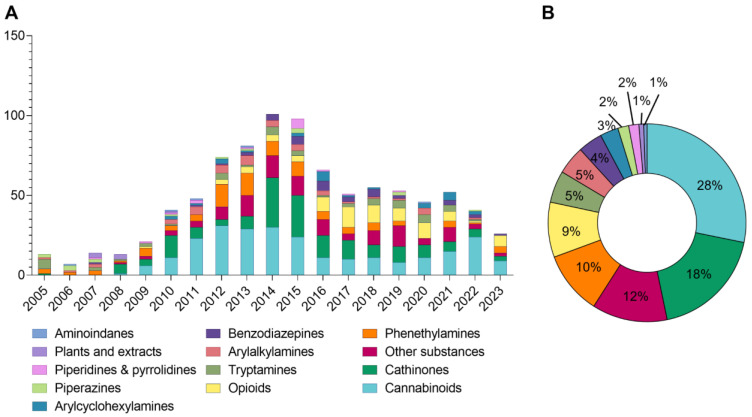
Number of new psychoactive substances (NPSs) reported, for the first time, to the EU Early Warning System, by category, from 2005 to 2023 (**A**) and total combined from 2005 to 2023 (**B**). Adapted from the European Drug Report of 2024 from EUDA [4].

The European Drug Report of 2024 [4] reported that the European Union Drugs Agency (EUDA) was monitoring around 950 NPSs at the end of 2023, 26 of them first reported in Europe in that year (Figure 1A). The two groups of NPSs most described over the years are synthetic cannabinoids and synthetic cathinones, which comprise, respectively, 28% and 18% of NPSs reported for the first time between 2005 and 2023 (Figure 1B) [4].

Synthetic cathinones are a class of β-keto phenethylamine derivatives originating from *S*-cathinone, an alkaloid found in khat (*Catha edulis*). They share structural and pharmacological similarities with amphetamine [5,6]. Derivatives of cathinone were first synthesized for therapeutic purposes, being the first methcathinone and mephedrone created at the end of the 1920s as potential antidepressants due to their stimulant properties to the central nervous system (CNS), which are associated with their interaction with monoamine transporters, namely dopamine (DA), norepinephrine (NE), and serotonin (SER) transporters (DAT, NET, and SERT, respectively) [7]. Afterwards, these compounds were found to have addictive properties and quickly became the focus of clandestine laboratories [8], emerging at the beginning of the 2000s, online and in smartshops, as bath salts under names like “Explosion”, “Bloom”, “Vanilla Sky”, “Bliss”, “Blue Silk”, and many others [9,10].

Synthetic cannabinoids, often described as synthetic cannabinoid receptor agonists (SCRAs), are structurally a diverse group that target the endocannabinoid system by interacting with two subtypes of cannabinoid receptors, CB1 and CB2, similarly to tetrahydrocannabinol (Δ^9^-THC), the main active compound in cannabis, and endogenous ligands such as the endocannabinoids anandamide and 2-arachydonylglycerol [11]. Synthetic cannabinoids were first produced in research labs with the goal of investigating their binding sites to explore the endocannabinoid system and to create alternatives for medicinal use without adverse effects [12,13]. Due to their similar properties to Δ^9^-THC often being more potent, clandestine laboratories began using the research on synthetic cannabinoids to create alternatives to marijuana. These synthetic cannabinoids started to emerge in the 2000s and were legally sold online or in smartshops under names such as “Spice”, “K2”, or “synthetic marijuana” [14,15].

After an abrupt increase in abuse cases with synthetic cathinones and synthetic cannabinoids, legislative control measures were created and the consumption of this first generation of NPSs became prohibited in several countries [10]. However, to circumvent the law, clandestine laboratories began to synthesize new derivatives. Therefore, every year, novel synthetic cathinones and synthetic cannabinoids emerge on the drug market, with their unknown properties still being a huge problem worldwide [16,17]. Although NPSs are widely studied, due to their structural diversity and the continuous emergence of novel compounds, new research studies are needed to elucidate their mode of action and assess their toxicological effects.

Metabolomics is an emerging field of study dedicated to identifying and quantifying all metabolites within a specific biological sample. This is achieved through advanced techniques such as mass spectrometry (MS) and nuclear magnetic resonance (NMR) spectroscopy, which provide a comprehensive snapshot of the metabolic state and pathways in living organisms [18]. Metabolomics has been applied to several xenobiotics (pesticides, pollutants, and carcinogens, among others) for the identification of toxicity-associated pathways, evaluation of cellular and organ toxicity, discovery of new biomarkers of effect or exposure, and development of toxicity prediction models, with the aim of a deeper understanding of how these xenobiotics exert their effects [19,20]. While its application to drugs of abuse remains relatively underexplored, metabolomics is a promising tool to clarify toxicity mechanisms, investigating toxicological effects and identifying biomarkers of exposure or effects associated with drugs of abuse, including NPSs [21,22].

Herein, a systematic review was performed, focusing on the use of metabolomics to examine the metabolic signature of synthetic cathinones and synthetic cannabinoids and evaluate their impact on cellular metabolism. This review aims to identify the main metabolic pathways and metabolites disrupted by synthetic cathinones and synthetic cannabinoids, assess analytical techniques and metabolomic strategies employed in the existing reports, evaluate the types of models and samples most used and other experimental conditions selected, compare results from studies with similar drugs, and highlight information available in these studies related to the mechanisms of action and toxicological effects of these compounds.

## 2. Literature Research Methodology

This research was structured in accordance with the Preferred Reporting Items for Systematic Reviews and Meta-Analyses (PRISMA) guidelines [23]. To select the relevant papers, research was conducted through the PubMed and SCOPUS databases considering the following keywords: “cathinones AND metabolomics” and “synthetic cannabinoids AND metabolomics”, searched in “Article title, Abstract, Keywords”. The selection of studies took place in September of 2024, with all papers being published within the time frame of 2012–September of 2024, since the first article found through this research was from 2012. The selection focused exclusively on English-language studies. After the identification of 90 records, they were first analyzed using Endnote 21 software to remove duplicates. During the screening phase, 63 records were screened by title/abstract to remove articles not relevant and review papers. In the second phase of the screening phase, a full-text review of 39 records was performed. Studies about the biotransformation of the compounds were excluded in this phase. Studies found through other methods, such as citation searching, were also included.

Screening at both the title/abstract and full-text stages and data collection were conducted first by one reviewer. All screening and data collection were further discussed and reviewed by the other reviewers. No automation tools were used. All collected data were analyzed critically and impartially following the guidelines of PRISMA.

Since this review focused on metabolomics-based studies investigating the effects of synthetic cathinones and synthetic cannabinoids on cellular metabolism, the primary outcomes sought in data collection included alterations in metabolic pathways and the identification of disrupted metabolites and affected biological systems (neuronal, hepatic, and renal, among others). Results from all relevant measures and analysis were included to allow a comprehensive coverage. If studies reported distinct outcomes or incomplete results, the data selected for analysis were based on their relevance to the aims of this review and consistency with other findings.

For the synthesis and presentation of results, a table was prepared including all studies with synthetic cathinones and synthetic cannabinoids. Studies with the same compound were grouped together and metabolomic approaches, techniques, samples, doses/concentrations, exposure time, and number of replicates were retrieved from each report and added to the table. The structure of each compound was created using ChemDraw 18.0. A summary of the main findings of each report was included in the table related to the number or the name of the most relevant disrupted metabolic pathways/metabolites and, if available, what type of alterations were observed (increase/decrease).

Doughnut charts illustrating the distribution of NPS classes, analytical techniques, metabolomic approaches, and types of samples/models employed in the metabolomic studies with synthetic cathinones and cannabinoids were created using GraphPad Prism 9. No further statistical analysis or meta-analysis was performed with any of the data.

The main findings of each report were analyzed and further discussed based on different topics that were divided into subsections: techniques, metabolomic approaches, types of samples or models, elucidation of toxicological effects, comparison with other drugs of abuse, influence of dose/concentration, metabolic pathways most affected by synthetic cathinones and synthetic cannabinoids, and limitations. Representative examples from the articles for each topic were described. Additionally, to illustrate some examples, two figures were created in BioRender. No meta-analysis was performed.

While a formal risk of bias assessment was not performed, we acknowledge that bias may exist in the studies reviewed due to differences in study design, sample selection, data collection methods, or statistical analyses. The possibility of publication bias, where studies with significant or positive results are more likely to be published, is acknowledged as a limitation.

Several measures were performed to minimize potential biases. The literature search was performed through multiple databases (PubMed and SCOPUS) and included citation searches to compile as many relevant studies as possible. Only original research articles addressing metabolomic studies of synthetic cathinones and synthetic cannabinoids were included, excluding review articles to avoid duplication of findings.

## 3. Results

**Figure 2:**
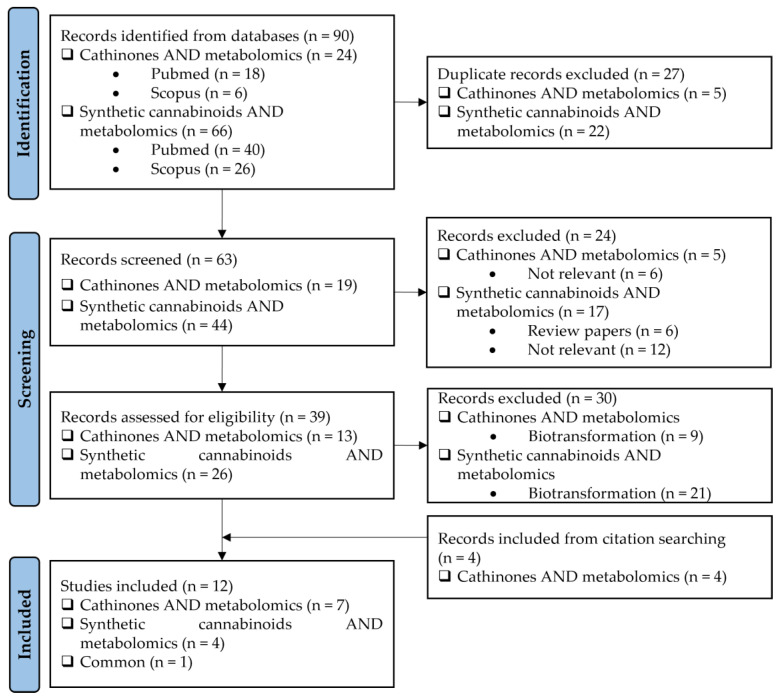
Flow diagram of literature search based on PRISMA guidelines (n = number of scientific articles; time frame: 2012–September 2024; database: PubMed and SCOPUS).

The PRISMA approach employed was performed according to the flowchart presented in Figure 2. In this search through PubMed and SCOPUS, 90 records were first identified. After the removal of duplicates, 63 records were screened by title/abstract to remove articles not relevant and review papers. A full-text review of 39 records was performed and studies about the biotransformation of the compounds were excluded in this phase. Although some of these records represented metabolomic studies with NPSs, biotransformation studies were out of the scope of this review. For instance, Manier et al. [24] employed an untargeted metabolomics workflow to study the *in vitro* biotransformation of the synthetic cathinones α-PBP and α-PEP in pooled human liver microsomes (pHLMs). Similarly, Kim et al. [25] used targeted and untargeted metabolite identification techniques to study the metabolomic effects of the synthetic cannabinoid MAM-2201 in human, mouse, and rat hepatocytes.

In the PRISMA search, studies found through other methods, such as citation searching, were also included. In the end, 12 scientific articles were selected for further discussion.

Relevant information about the articles is summarized in Table 1.

## 4. Discussion

**Figure 3:**
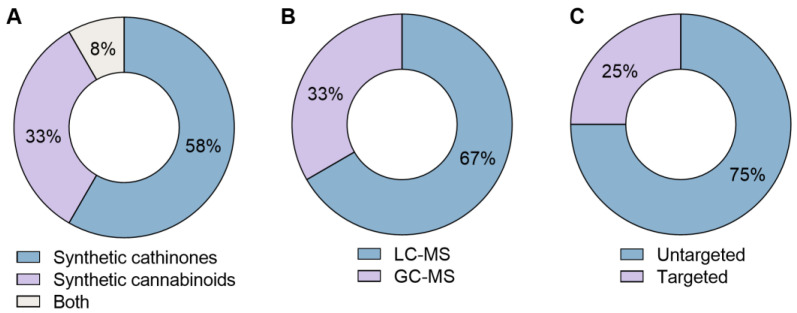
Doughnut charts illustrating the distribution of NPS classes (**A**), analytical techniques (**B**), and metabolomic study approaches that employed synthetic cathinones and cannabinoids (**C**).

Twelve articles were selected through PRISMA-based review, focusing on metabolomic studies involving synthetic cathinones and synthetic cannabinoids. The majority of the studies (58%) addressed synthetic cathinones (Figure 3A). One of the articles included compounds from both classes [30].

### 4.1. Techniques

In metabolomics research, common techniques include NMR spectroscopy and MS, typically paired with gas chromatography (GC) or liquid chromatography (LC). NMR provides several advantages, such as the ability to analyze a wide variety of sample types without destroying them, as well as requiring minimal sample preparation [37,38]. Additionally, its high reproducibility and potential for automation makes NMR well suited for large-scale, high-throughput metabolomics applications [39]. Nonetheless, NMR has some disadvantages, which include a lower sensitivity, reduced specificity, and a limited range of detectable metabolites when compared to MS [38,40].

On the other hand, MS offers higher sensitivity and specificity, allowing the detection of a broader spectrum of metabolites, which makes it especially useful for studying complex biological samples. However, MS typically requires coupling with chromatographic techniques such as LC, GC, or, although less applied, capillary electrophoresis (CE). Some disadvantages of MS include its lower reproducibility and inability to recover samples [38,41]. Regarding the techniques used in the reported studies (Figure 3B), only MS techniques were reported, with LC-MS being most employed over GC-MS (67% for LC-MS vs. 33% for GC-MS); none of the studies utilized NMR. In fact, MS is the most reported technique in metabolomics due to its superior sensitivity, high throughput capability, and its ability to detect a greater number of metabolites in complex biological samples, explaining the preference for MS over NMR in these studies [42]. Moreover, when compared to LC-MS, GC-MS presents lower costs, better reproducibility, and more extensive mass spectral libraries. However, GC-MS is limited to volatile compounds or those with a need for derivatization treatment, requiring more extensive sample preparation and often longer analysis [41,42]. Additionally, ultra-high-performance liquid chromatography (UHPLC) coupled with MS enhances separation, sensitivity, and peak capacity [42].

### 4.2. Metabolomic Approaches

Regarding metabolomics strategies, they are generally categorized into two main types: untargeted and targeted approaches [43,44]. Untargeted metabolomics is often the starting point in research, as it allows for a broader detection and identification of metabolites within a sample, without prior knowledge of the metabolome. This approach enables the investigation of complex metabolic networks and the relationships between various metabolites [43,45]. In contrast, the targeted approach focuses on a specific set of metabolites, typically for quantification purposes. It is often used to confirm results from an untargeted analysis and to identify potential biomarkers of interest [44]. Most studies selected above employed untargeted approaches (75%). It was found that only 25% of the articles used targeted approaches (Figure 3C), being primarily focused on studies involving synthetic cannabinoids.

As an example, in a study by Markin et al. [34] (Table 2), 36 metabolites associated with neurotransmission were quantified.

Additionally, Olesti et al. [30] investigated both a synthetic cannabinoid (JWH-018) and a synthetic cathinone (mephedrone), examining the levels of 168 markers in rat samples. These markers included metabolites from serotonergic, dopaminergic, and noradrenergic pathways, as well as corticosteroids and sexual hormones. Since both synthetic cathinones and synthetic cannabinoids exert neuronal effects [13,46], focusing metabolomics studies on neurotransmission-related metabolites can provide valuable insight into their mechanisms of action.

Nonetheless, although targeted analysis provided detailed information on specific metabolic pathways, untargeted analysis was preferred, since it gives an overview of metabolic changes and can reveal other pathways that are linked to the toxicological action of drugs [29].

### 4.3. Types of Models and Samples

**Figure 4:**
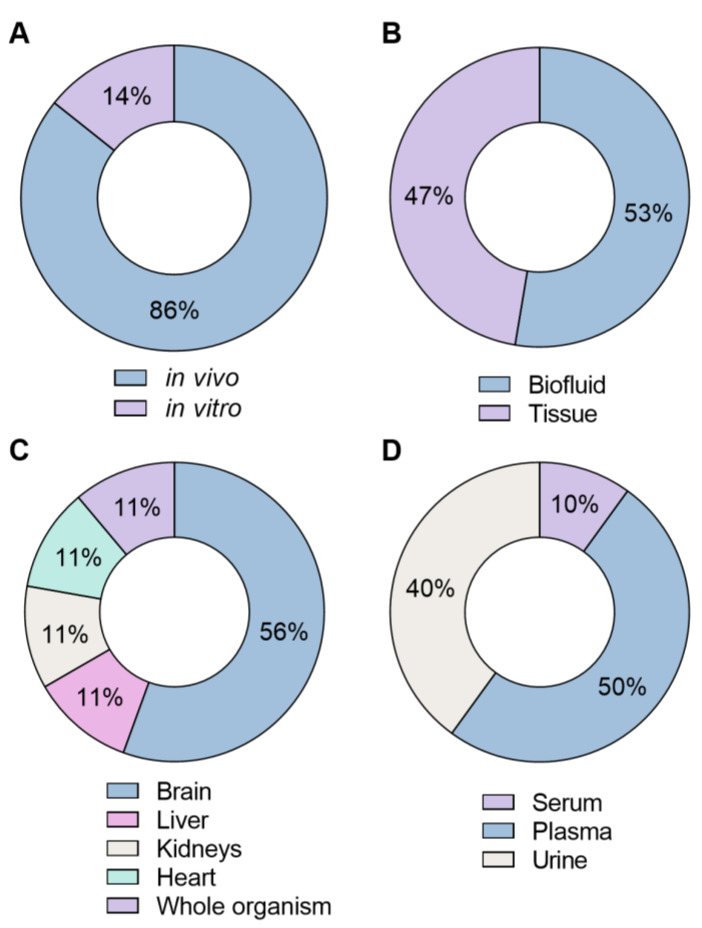
Doughnut charts illustrating the distribution of the type of samples/models employed in the metabolomic studies with synthetic cathinones and cannabinoids. (**A**)—*in vivo* vs. *in vitro*; (**B**)—tissue vs. biofluids; (**C**)—types of tissues; and (**D**)—types of biofluids.

Regarding the type of model selected for these studies, *in vivo* models were preferred over *in vitro* models, corresponding to 86% of the studies (Figure 4A). *In vivo* studies are considered more specific and reliable, as they are performed in a living organism, providing a holistic understanding of complex biological interactions. Animal models such as rat, mouse, rabbit, and zebrafish are commonly used to predict human metabolic responses. However, *in vivo* models are often more expensive, time-consuming, and subjected to ethical considerations and regulatory requirements. Moreover, there are significant physiological differences between humans and animals and species-specific pathways that can affect the extrapolation of results [47]. On the other hand, *in vitro* models are easier to manage, less costly, and provide more straightforward data interpretation. Nevertheless, they do not represent the complexity of an organism in its natural environment, as they are a simplified model maintained under artificial culture conditions. To improve extrapolation validity, complementary studies using *in vivo* models, *in vitro* human-derived models, or *ex vivo* human tissues should be performed. Combining information from multiple models can enhance the accuracy and translational relevance of metabolomic findings [48,49]. For instance, previous *in vitro* studies with primary rat hepatocytes [50] and HepG2 [51] showed that the synthetic cathinone MDPV induces a decrease in hepatic glutathione (GSH). Araújo et al. [22] also described that MDPV led to a decrease in metabolites associated with GSH in liver samples, correlating with the previous findings. Moreover, both the decrease in metabolites related to GSH and ascorbic acid suggested an impairment of antioxidant defenses system, consistent with the metabolic profile observed in primary mouse hepatic cells after exposure to MDPV [22].

Ideally, human samples would be preferred for investigating the human metabolome. Studies with human samples, such as plasma, urine, and brain tissues, provide more direct information regarding the metabolic disruptions and toxicological effects of NPSs, which can be useful for the identification of biomarkers of exposure and the development of therapeutic treatments. Although other *in vivo* models offer valuable information, they have several limitations, as previously mentioned. Thus, the use of human samples is critical to understand the impact of NPSs on human health, since they provide the most direct and accurate results. However, for NPSs and other drugs of abuse, acquiring human samples requires controlled drug administration studies, which are scarce due to ethical restrictions in many countries [29]. Among the compiled articles, only Steuer et al. [29] used human plasma samples to perform a comparative analysis of the metabolic effects of MDMA, amphetamine, and mephedrone.

Sample selection is crucial, and should align with the aim of the study, as distinct samples can provide diverse information. In this compilation, it was found that, although with similar percentages, biofluids were preferred over tissue samples (53% for biofluids vs. 43% for tissues) (Figure 4B). Among tissues samples, brain samples were the most reported, with 56% (Figure 4C), while for biofluids, plasma was preferred, with 50% of articles, followed by urine with 40% (Figure 4D). Synthetic cathinones and synthetic cannabinoids exert neuronal effects by interacting with monoamine transporters and cannabinoid receptors [13,46]. While urine and blood metabolomics can provide a comprehensive view of the metabolomic changes in the organism, the analysis of brain tissue offers more specific insight into the mechanism of action [52,53]. For instance, Li et al. [35] selected the mouse hippocampus to evaluate the metabolic effects of the synthetic cannabinoid JWH-018. This choice was based on the fact that the CB1 receptor is predominantly expressed in the hippocampus, a brain region crucial for learning and short-term memory [35].

A GC-MS metabolomics approach was applied by Araújo et al. [22] to analyze the metabolic effects of MDPV in several organs and urine of mouse. Brain, kidneys, heart, and liver were selected as the target organs, since MDPV was shown to induce severe multi-organ dysfunction affecting mostly these organs. The urine metabolome was also evaluated due to its ability to provide a more comprehensive overview of the general metabolic processes that take place over time in the organism. Fifty-eight metabolites in the liver, 23 metabolites in the kidneys, 31 metabolites in the brain, 14 metabolites in the heart, and 32 metabolites in urine were identified related to potential cell disturbance caused by MDPV [22].

The metabolic profiles of the liver and kidney were the most affected by MDPV, while the heart and brain were the least affected. Overall, each organ displayed a unique metabolic profile, but energy metabolism and oxidative stress seem to be the metabolic pathways that contribute the most to the toxic effects of MDPV [22].

In a study described by Olesti et al. [30], brain, plasma, and urine samples were collected from rats to evaluate the metabolic effect of several drugs of abuse, including mephedrone and JWH-018, two NPSs. As previously mentioned, this study followed a targeted approach to analyze the levels of metabolites related to neurotransmitter systems and steroid hormones; brain samples were the ideal choice. However, brain samples can only be collected postmortem. Thus, the establishment of methods using non-invasive samples, such as blood and urine, for the determination of brain-related metabolites that correlate with results obtained from the brain samples is extremely important [35]. In urine samples, only monoamine neurotransmitter levels were evaluated, while in plasma samples, only steroid hormones levels were analyzed. When compared with the metabolomic results from the brain samples, the changes in steroid hormones in plasma were highly similar, while the levels of monoamine neurotransmitters in urine had no correlation with brain results. This result suggested that plasma analysis could be applied to predict steroid hormone levels in rat brain after the administration of several drugs of abuse [30].

Shestakova et al. [33] and Markin et al. [34] evaluated the metabolic effects of the synthetic cannabinoid 5F-APINAC in rabbit and zebrafish, respectively. In both studies, metabolites associated with the kynurenine pathway were affected. The perturbation of this metabolic pathway had the most pronounced effects in rabbit plasma samples. In contrast, while other neurotransmitter systems, such as the γ-aminobutyric acid (GABA)/Glu, dopaminergic/adrenergic, and cholinergic neurotransmitter systems, remained unchanged in rabbit plasma, they were disrupted in zebrafish. This could be attributed to physiological differences between the two organisms or, alternatively, to the fact that in zebrafish, the entire organism was analyzed, reflecting the metabolic changes across all organs, whereas in rabbit, only plasma samples were examined [33,34].

Only 14% of the articles employed *in vitro* models, all focused on synthetic cathinones with hepatic cellular models. Specifically, two studies used primary rat hepatocytes to assess the metabolic effect of MDPV [28] and methylone [32], while another utilized HepaRG cells to investigate the metabolism of α-PBP and α-PEP [31]. The rationale for selecting these models is based on the liver being a primary site of synthetic cathinone toxicity, combined with the limited understanding of the mechanisms underlying their hepatotoxic effects [28].

### 4.4. Elucidation of Toxicological Effects

Metabolomics can be used to better elucidate the mechanisms of action of drugs of abuse, including NPSs, and to explain the toxicological consequences observed for users, which is crucial given the rapid emergence of NPSs and their significant public health risks. Using information regarding the disrupted metabolic pathways can give valuable insights into the potential for organ damage, neurological effects, or systemic toxicity caused by synthetic cathinones and cannabinoids.

For instance, Araújo et al. [22] described that MDPV caused an increase in palmitoleic and oleic acid levels in mouse liver, suggesting an intensification of the biosynthesis of unsaturated fatty acids to obtain energy. Previous histopathological analysis showed hepatic macrovesicular steatosis caused by MDPV, which could be, in part, caused by the upregulation of this pathway. Moreover, lactic acid was augmented in the renal metabolic profile, which could increase the acidity of the microenvironment, contributing to the metabolic acidosis reported in MDPV intoxications. It has been reported that, in metabolic acidosis conditions, the kidneys accumulate amino acids from the blood [54]. In this study, the levels of several amino acids were also increased in the kidney samples. Additionally, the metabolic profile of the heart revealed an increase in fatty acid biosynthesis, which can be attributed to the heightened demand for adenosine triphosphate (ATP) production through fatty acid β-oxidation. This metabolic shift is likely a response to the elevated cardiac workload from MDPV-induced tachycardia [22].

**Figure 5:**
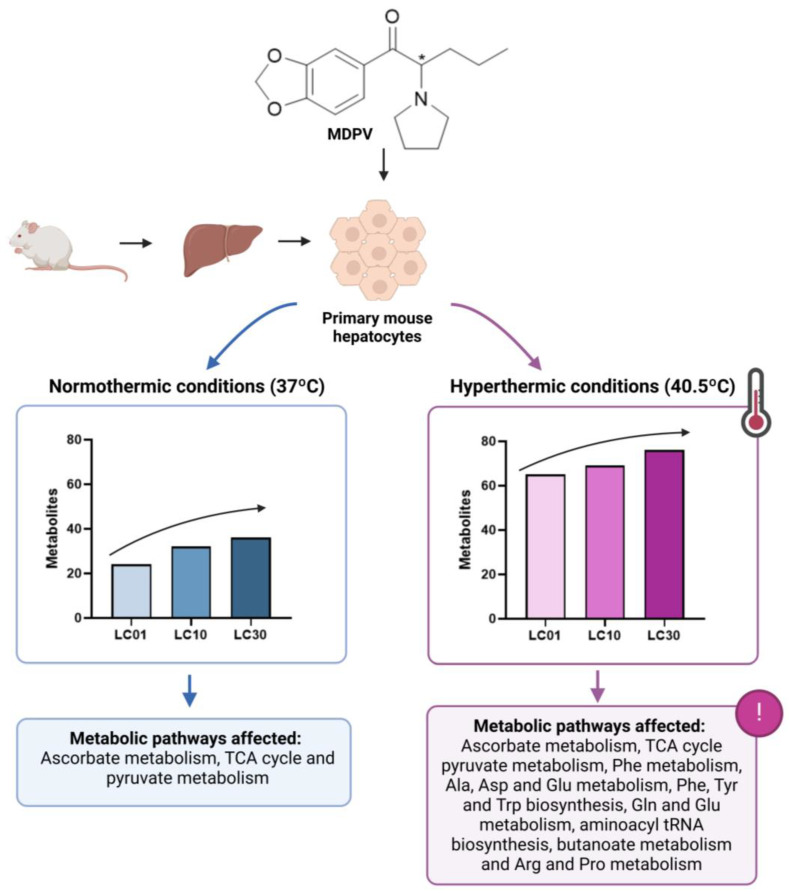
The number of metabolites and metabolic pathways found altered in MDPV-exposed primary mouse hepatocytes under normothermic and hyperthermic conditions. Created in BioRender. Almeida, A. (2024) https://BioRender.com/o94v700. TCA: Tricarboxylic acid; *: Chiral center.

In another study by Araújo et al. [28], the effect of MDPV on the metabolic profile of primary mouse hepatocytes, under normothermic (37 °C) and hyperthermic (40.5 °C) conditions, was evaluated (Figure 5). The comparison between these conditions was performed since human case reports have shown that one of MDPV’s most dangerous effects is the elevation of body temperature to values above 41 °C. Moreover, the liver is extremely affected by hyperthermic conditions and, in primary rat hepatocytes, MDPV displayed higher hepatoxicity under hyperthermic conditions. Thus, understanding the underlying metabolic pathways affected by MDPV in both conditions is important. The metabolic systems affected under normothermic conditions, such as ascorbate metabolism, the TCA cycle, and pyruvate metabolism, were significantly enhanced under hyperthermic conditions. Additionally, other metabolic pathways not affected under normothermic conditions were affected under hyperthermic conditions, namely Asp and Glu metabolism, Phe and Tyr biosynthesis, aminoacyl-tRNA biosynthesis, butanoate metabolism, among others [28].

Li et al. [35] reported that JWH-018 induces a significant increase in the levels of endocannabinoids such as anandamide and 2-arachidonoylglycerol in the hippocampus of mice. These endogenous compounds serve as natural ligands for the CB1 receptor. Elevated levels of endocannabinoids are associated with the suppression of synaptic long-term potentiation, a crucial process for memory impairment [55]. This finding aligns with the well-known established link between synthetic cannabinoids and memory impairment [56]. Therefore, this result suggested that the increase in the two endocannabinoids might be related to the memory impairment caused by JWH-018. Moreover, the alteration of these endocannabinoids could indicate their use as biomarkers of abuse of JWH-018 or other related synthetic cannabinoids [35].

In Markin et al.’s study [34], 5F-APINAC decreased the GABA levels in zebrafish at the highest concentration tested. GABA is an inhibitory neurotransmitter associated, for instance, with the regulation of anxiety. Cannabinoids have been reported to inhibit the release of GABA and inhibit the GABAergic innervation system, as highlighted in recent studies [57]. Moreover, studies have indicated that the endocannabinoid system plays a role in modulating GABA receptors [58]. Given that synthetic cannabinoids affect the endocannabinoid system, it could be expected that GABA levels are altered by them [34].

Having a better understanding of the toxicological effects of NPSs highlights their potential life-threatening outcomes, which could further be used to predict adverse effects after NPS consumption and improve intoxication treatment.

### 4.5. Comparison with Other Drugs of Abuse

**Figure 6:**
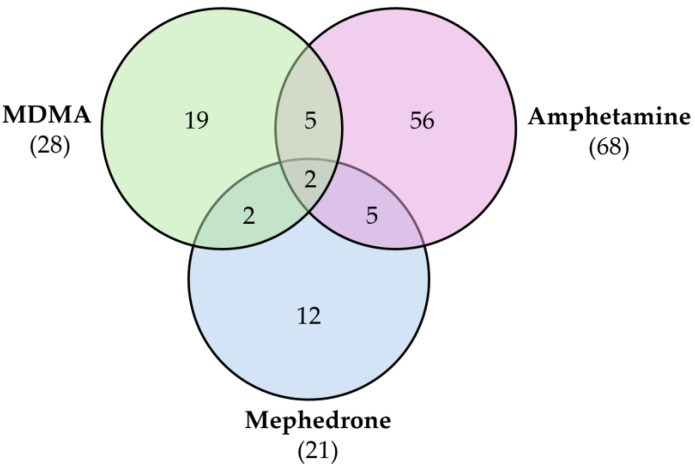
Venn diagram of metabolites altered by MDMA, amphetamine, and mephedrone in human blood samples. Adapted from [29].

Synthetic cathinones and amphetamines are structurally similar, the difference being the β-keto group present in cathinones, which increases their polarity, often decreasing their potency [59]. Moreover, these two groups are also functionally similar since their psychostimulant properties arise from their effects on monoamine transporters [46]. Thus, similarities could be expected in their metabolic profiles. Some studies focused on the comparison between the two groups. For instance, as previously mentioned, Steuer et al. [29] performed a comparative metabolomics study with MDMA, amphetamine, and mephedrone in humans (Figure 6). Amphetamine showed a higher affinity to DAT and NET, increasing the release of DA and NE while MDMA displayed a higher affinity to SERT, inducing the release of SER. Mephedrone has similarities with both since it acts in DAT and SERT. The results of this work showed that mephedrone’s effects on the metabolic profile are more related to amphetamine than MDMA [29].

Araújo et al. [32] evaluated the metabolic profile of primary mouse hepatocytes after exposure to methylone. At a subtoxic concentration, there was an increase in the levels of malate and fumarate, two TCA cycle intermediates, and in the levels of glucogenic amino acids, with this effect being enhanced at higher concentrations [32]. When compared with a previous work with MDMA under the same conditions, although MDMA and methylone both affected the levels of amino acids and TCA intermediates, they had opposite effects, with the levels being significantly reduced after MDMA exposure [60].

Furthermore, in the metabolic profile of mouse heart after exposure to MDPV, there was an increase in fatty acid metabolism, which was also observed in a previous study with MDMA [22,61].

Synthetic cannabinoids target the endocannabinoid system by interacting with cannabinoid receptors, CB1 and CB2, similarly to other natural cannabinoids such as Δ^9^-THC and endogenous endocannabinoids [11]. Therefore, similarities could be expected in their metabolic profiles.

Markin et al. [34] evaluated the metabolic profile of zebrafish after exposure to 5F-APINAC. An increase in DA levels was observed after both acute and chronic exposure, with a slight decrease in chronic exposure at the highest doses [34]. The endocannabinoid system modulates DA receptors. Given that synthetic cannabinoids affect the endocannabinoid system, it could be expected that DA levels are affected by them. Furthermore, a previous work showed that acute Δ^9^-THC exposure resulted in an increase in dopaminergic cell firing, DA synthesis, and DA release [62]. Another work with Δ^9^-THC and WIN 55,212-2 also reported an increase in dopa and NE synthesis in rat brain [63]. Thus, 5F-APINAC seems to have an influence on the CNS through the dopaminergic system. Additionally, significant changes in Trp levels were observed. The previous work with Δ^9^-THC and WIN 55,212-2 reported the inhibition of the synthesis of 5-hydrotryptophan and SER through influence on Trp hydroxylase, which could lead to an accumulation of Trp [63].

Zaitsu et al. [36] reported the effects of MAM-2201 on TCA cycle intermediates in the metabolic profile of rat cerebrum, suggesting a disruption of energy metabolism. A similar energy metabolism disruption was observed in a previous work with pancreatic cancer cells by the synthetic cannabinoids arachidonyl cyclopropamide and GW405833 by the inhibition of α-ketoglutaric acid dehydrogenase [64]. Similarly, it was suggested that the decrease in malic acid and succinic acid levels could be influenced by the inhibition of this enzyme [36].

The comparison of NPSs with other drugs of abuse through metabolomics offers significant value for public health, allowing, for instance, the prediction of adverse effects and development of improved diagnostic tools. Therefore, the dangers posed by NPSs can be better understood and their impact on individuals and the general public can be more easily mitigated.

### 4.6. Influence of Dose/Concentration

The metabolic profile of an organism can be influenced by the dose or concentration of the compound to which it is exposed, as well as by whether the exposure is acute or chronic. At subtoxic concentrations of methylone [32] and MDPV [28], several metabolic pathways were affected in primary mouse hepatocytes. For instance, MDPV at a lower subtoxic concentration (LC01) induced significant alterations in 24 metabolites under normothermic conditions, with ascorbate metabolism being one of the most significantly altered metabolic pathways. An increase in concentration (LC10) led to significant effects on 32 metabolites, which apart from ascorbate synthesis, were related to the TCA cycle and pyruvate metabolism. Furthermore, toxic concentration (LC30) affected 36 metabolites (LC30) from the same metabolic pathways, but with aggravated effects [28].

Methylone at LC01 induced effects on metabolic systems related to the urea cycle, the TCA cycle, and aspartate metabolism. A higher concentration (LC10) led to effects on six metabolic pathways, namely Phe and Tyr metabolism, the malate–Asp shuttle, Glu metabolism, glutathione metabolism, Cys metabolism, and the urea cycle. Interestingly, some of these metabolic alterations seem to be related to initial protective mechanisms, such as the upregulation of TCA cycle intermediates and an increase in the levels of metabolites involved in glutathione synthesis, Cys, and Glu, which were suggested to be an initial protective response to restore energy and protection against oxidative stress, respectively [32].

**Figure 7:**
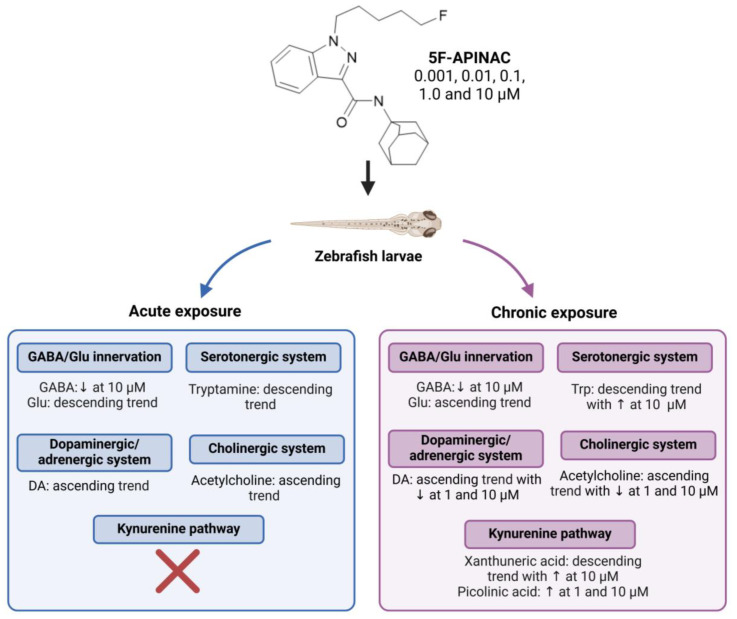
Summary of the effects of acute or chronic exposure to 5F-APINAC on metabolites related to neurotransmitter pathways in zebrafish. Created in BioRender. Almeida, A. (2024) https://BioRender.com/i68o167. DA: dopamine; GABA: γ-Aminobutyric acid; ↑: Increase ↓: Decrease.

Markin et al. [34] evaluated the effects of short- and long-term exposure of different concentrations of 5F-APINAC (0.001, 0.01, 0.1, 1.0, and 10 µM) to zebrafish larvae/eggs (Figure 7). Similar metabolic pathways were disrupted by the time conditions, such as the GABA/Glu, serotonergic, dopaminergic/adrenergic, and cholinergic systems, with some differences between the effects on metabolites. For instance, in the GABA/Glu system, with the increase in concentration, Gln decreased for acute exposure and increased for chronic exposure. In the serotonergic system, Trp levels were only disrupted in chronic exposure while tryptamine was only affected in acute exposure. Moreover, the kynurenine pathway was only affected by chronic exposure, with effects on the levels of xanthurenic and picolinic acid [34].

### 4.7. Metabolic Pathways Most Affected by Synthetic Cathinones and Synthetic Cannabinoids

Overall, studies on synthetic cathinones commonly reported disruptions in metabolic pathways related to energy metabolism, including amino acids synthesis and metabolism, the TCA cycle, and pyruvate metabolism. These effects appear consistently across various conditions and distinct synthetic cathinones. Moreover, some studies highlight disruptions in pathways associated with antioxidant defenses, such as Glu, Cys, and glutathione metabolism, as well as lipid biosynthesis and metabolism.

For synthetic cannabinoids, the literature on metabolomic studies is still extremely scarce. Among the five studies reported, three of them use targeted metabolomics to investigate the effects of the compounds in the neurotransmission process [30,33,34]. Additionally, two of these studies tested the same compound, 5F-APINAC, in rabbit plasma [33] and zebrafish larvae/eggs [34]. The kynurenine pathway was the only metabolic pathway commonly affected in both studies. In the other targeted approach, the effects of JWH-018 on the metabolic profile of rat brain were evaluated [30]. Compared with the studies with 5F-APINAC, only the dopaminergic system pathway was found to be affected in the study with zebrafish. Moreover, in the two studies based on untargeted approaches with MAM-2201 [36] and JWH-018 [35] in rat brain, some similarities were found in the metabolomic profile, such as effects on metabolites related to the TCA cycle and amino-acid related pathways, which, similarly to synthetic cathinones, are associated with disruption in energy metabolism. Additionally, Glu was significantly affected in both works. Interestingly, Markin et al. [34] described that the GABA/Glu system was also significantly disrupted by 5F-APINAC in zebrafish. However, the metabolites affected were GABA and Gln, and no effect was found in the levels of Glu. Nonetheless, the consistent correlation observed across the three studies hints at the potential involvement of this metabolic pathway in mediating the effects of synthetic cannabinoids.

Furthermore, these and future metabolomic findings could be used to identify biomarkers of exposure to or the toxicity of NPSs, allowing the development of diagnostic tools for better NPS detection. This could not only be used for a quicker response in the treatment of an intoxication but also to closely monitor trends in NPS consumption, leading to quicker responses to emerging threats.

### 4.8. Limitations

Metabolomic studies with synthetic cathinones and synthetic cannabinoids are still extremely scarce, which, along with the variability among the experimental conditions and analytical techniques in the included studies, complicates direct comparisons and the generalization of the results. Furthermore, ethical and practical constraints on human studies lead to the use of animal and cellular models, which might not fully replicate the human metabolome.

Due to the heterogeneity in study conditions, outcomes, and the nature of this review, no meta-analysis was conducted; thus, no quantitative comparisons were made. Lastly, potential publication bias favoring studies with significant results may have influenced the overall findings.

These limitations highlight the importance of future research in this emerging field to address these gaps more comprehensively.

## 5. Conclusions

This review compiles metabolomic studies investigating the effects of synthetic cathinones and synthetic cannabinoids on cell metabolism, encompassing 12 selected papers: 7 focused on synthetic cathinones, 4 on synthetic cannabinoids, and 1 addressed both groups. The main technique applied in these studies was LC-MS following untargeted approaches. Regarding the types of model/samples, the *in vivo* models were the most reported. Although research in this area remains limited, metabolomics has demonstrated its value as a powerful tool to elucidate the mechanism of action of these two groups of NPSs and to uncover the metabolic alterations underlying their toxicological effects. Moreover, both groups seem to disrupt mostly pathways related to energy metabolism. Considering the continuous growing in the number of NPSs every year and their dangers to public health, more studies should be developed in this area.

## Figures and Tables

**Table 1:** Summary of metabolomic studies reported in the literature regarding synthetic cathinones and synthetic cannabinoids.

Compound	Experimental Conditions	Main Results	Ref.
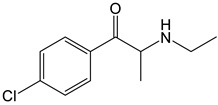 4-CEC	Approach: Untargeted Sample: Mouse serum Dose: 40 mg/kg Exposure time: 2 h or 7 days Replicates: 7 Technique: UHPLC-MS/MS	Metabolic pathways most affected: Trp metabolism, niacin and nicotinamide metabolism, Arg and Pro metabolism, and Arg biosynthesis Other metabolic pathways affected: Energy-related metabolic pathways such as Gly, Ser, and Thr metabolism, and glycerophospholipid metabolism 14 metabolites upregulated and 10 metabolites downregulated in the 2 h administration group	[26]
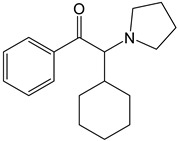 PCYP	Approach: Untargeted Sample: Rat urine and plasma Dose: 2 mg/kg body weight Exposure time: 1 h for plasma and 24 h for urine Replicates: 5 Technique: LC-HRMS	Rat plasma samples: Increase in adenosine, 3-methyladipic acid, and quinoline-2-ol Rat urine samples: Increase in quinoline-2-ol, daidzein, and dihydroxyquinoline Decrease in kynurenic acid	[27]
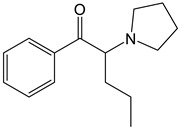 MDPV	Approach: Untargeted Sample: Mouse liver, kidneys, brain, heart tissues, and urine Dose: 2.5 mg/kg or 5 mg/kg every 2 h Exposure time: 24 h Replicates: 10 Technique: GC-MS	Metabolites and metabolic pathways affected in each sample: Liver: 58 metabolites Gly, Ser, and Thr metabolism, pantothenate and CoA biosynthesis, aminoacyl-tRNA biosynthesis, Val, Leu, and Ile biosynthesis, glutathione metabolism, biosynthesis of unsaturated fatty acids, nitrogen metabolism, and Cys and Met metabolism Kidneys: 23 metabolites Gly, Ser, and Thr metabolism, pantothenate and CoA biosynthesis, aminoacyl-tRNA biosynthesis, glutathione metabolism, nitrogen metabolism, Phe, Tyr, and Trp biosynthesis, cyanoamino acid metabolism, methane metabolism, Phe metabolism, beta-Ala metabolism, and Ala, Asp, and Glu metabolism Brain: 31 metabolites Aminoacyl-tRNA biosynthesis, Val, Leu, and Ile biosynthesis, nitrogen metabolism, butanoate metabolism, synthesis and degradation of ketone bodies, and d-Gln and d-Glu metabolism Heart: 14 metabolites Fatty acid biosynthesis Urine: 32 metabolites Gly, Ser, and Thr metabolism and pantothenate and CoA biosynthesis General results: Higher metabolic disturbance in liver and kidneys Each biological matrix exhibits a unique metabolic profile, often reflecting processes associated with oxidative stress and energy metabolism	[22]
	Approach: Untargeted Sample: Primary mouse hepatocytes Concentration: 70, 240, and 477 μM Exposure time: 24 h Replicates: 10 Technique: GC-MS	Normothermic conditions (37 °C) Metabolic pathways affected: ascorbate metabolism, TCA cycle, and pyruvate metabolism Hyperthermic conditions (40.5 °C) Enhanced effects on the same metabolic pathways Other metabolic pathways: Phe metabolism, Ala, Asp, and Glu metabolism, Phe, Tyr, and Trp biosynthesis, Gln and Glu metabolism, aminoacyl tRNA biosynthesis, butanoate metabolism, and Arg and Pro metabolism	[28]
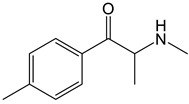 Mephedrone	Approach: Untargeted Sample: Human plasma Dose: 1.4 ± 0.2 mg/kg bodyweight Exposure time: 3 and 6 h Replicates: 5 Technique: UHPLC-TOF-MS	21 metabolites affected Pathways most affected: biosynthesis of unsaturated fatty acids, linoleic acid metabolism, steroid hormone biosynthesis, taurine and hypotaurine metabolism, aminoacyl-tRNA biosynthesis, and Arg and Pro metabolism Comparative study with MDMA and amphetamine: Metabolic responses more similar to those induced by amphetamine than by MDMA	[29]
	Approach: Targeted Sample: Rat urine, plasma and brain Dose: 30 mg/kg Exposure time: 1 or 4 h Replicates: 6 Technique: UHPLC-TQ-MS	Depleting effect on SER brain concentrations Upregulation of DA turnover Upregulation of NE levels and turnover of its metabolites in all brain areas in the first hour Increased release of progesterone and corticosteroids within the first hour, and androgens at 4 h	[30]
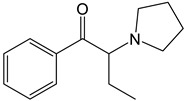 α-PBP	Approach: Untargeted Sample: HepaRG cells Concentration: 12.5 or 25 µM Exposure time: 24 h Replicates: - Technique: HPLC-HRMS/MS	Only *N*-methylnicotinamide significantly affected	[31]
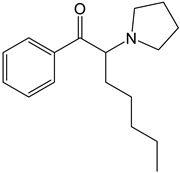 α-PEP (PV8)		Metabolites affected: cholesterol sulfate and 25-hydroxy cholesterol	
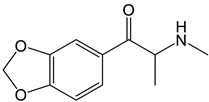 Methylone	Approach: Untargeted Sample: Primary mouse hepatocytes Concentration: 0.184 mM and 0.390 mM Exposure time: 24 h Replicates: 10 Technique: GC-MS	Metabolic pathways affected: Energy production processes: TCA cycle, amino acid metabolism, and pyruvate metabolism Cellular antioxidant defenses: Glu, Cys, and glutathione metabolism Hepatic enzymes: hydrocarbon, alcohol, aldehyde, and ketone metabolism	[32]
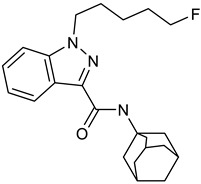 5F-APINAC	Approach: Targeted Sample: Rabbit plasma Concentration: 0.1, 1 and 2 mg/kg Exposure time: Nine time points (0–24 h) Replicates: 4 Technique: UHPLC-MS	Serotonergic system/SER pathway Trp decreased significantly immediately after administration but returned to normal after 6 h Asp innervation system Asp increased Kynurenine pathway L-Kynurenine, kynurenic acid, xanthurenic acid, and quinolinic acid increased Anthranilic acid decreased Microbial Trp catabolism Indole-3-propionic acid and indole-3-carboxaldehyde increased Indole-3-lactic acid decreased	[33]
	Approach: Targeted Sample: Zebrafish eggs/ larvae Concentration: 0.001, 0.01, 0.1, 1.0 and 10 μM Exposure time: 4 or 96 h Replicates: 20 Technique: UHPLC-MS/MS	GABA/Glu innervation GABA decreased at 10 µM Gln decreased in acute exposure and increased in chronic Serotonergic system/SER pathway Trp shows a descending trend during chronic exposure, with an increase observed at 10 µM. Tryptamine decreased for acute exposure Dopaminergic/adrenergic system DA exhibits an ascending trend during acute exposure, while chronic exposure shows an initial increase followed by a decrease at 1 µM and 10 µM concentrations Cholinergic system Acetylcholine exhibits an ascending trend during acute exposure, while chronic exposure shows an initial increase followed by a decrease at 1 µM and 10 µM Kynurenine pathway Xanthurenic acid shows a descending trend during chronic exposure, with an increase at 10 µM Picolinic acid increased at 1 and 10 µM Others Citrulline exhibits a descending trend during acute exposure, with a significant increase observed at 10 µM during chronic exposure	[34]
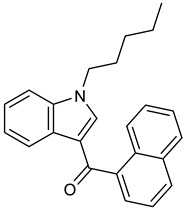 JWH-018	Approach: Untargeted Sample: Mice hippocamp Concentration: 1 mg/kg Exposure time: 2 h Replicates: 10 Technique: UHPLC-TOF/MS	Reduction in NAA Amino acids: Glu, Phe, and Tyr increased TCA cycle: Succinic acid increased Fatty acid metabolism: AEA and 2-AG increased	[35]
	Approach: Targeted Sample: Rat urine, plasma, and brain Dose: 10 mg/kg Exposure time: 1 and 4 h Replicates: 6 Technique: UHPLC-TQ-MS	Increase in the turnover of the dopaminergic system Lower recovery of 3-methoxytyramine No effect on NE release Lower recovery of normetanephrine and metanephrine Minor changes in testosterone concentrations	[30]
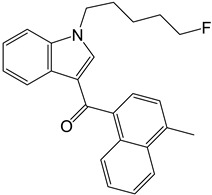 MAM-2201	Approach: Untargeted Sample: Rat cerebrum Dose: 5 or 15 mg/kg Exposure time: 2 h Replicates: 5 Technique: GC-MS/MS	12 metabolites altered TCA cycle: malic and succinic acid decreased Glu decreased Phe increased	[36]

4-CEC: 4-Chloroethcathinone; 2-AG: 2-Arachidonoylglycerol; 5F-APINAC: Adamantan-1-yl 1-(5-fluoropentyl)-1*H*-indazole-3-carboxylate; AEA: Anandamide; GC: Gas Chromatography; HRMS: High-resolution Mass Spectrometry; JWH-018: 1-Naphthalenyl(1-pentyl-1*H*-indol-3-yl)methanone; LC: Liquid Chromatography; MAM-2201: [1-(5-Fluoropentyl)-1*H*-indol-3-yl](4-methyl-1-naphthalenyl)methanone; MDPV: 3,4-Methylenedioxypyrovalerone; MS: Mass Spectrometry; NAA: *N*-Acetyl-L-aspartate; NE: Norepinephrine; PCYP: 2-Cyclohexyl-1-phenyl-2-(1-pyrrolidinyl)-ethanone; SER: Serotonin; TCA: Tricarboxylic acid; TOF: Time Of Flight; TQ: Triple Quadrupole; UHPLC: Ultra-High-Performance Liquid Chromatography; α-PBP: α-Pyrrolidinobutiophenone; α-PEP: α-Pyrrolidinoheptaphenone.

**Table 2:** Metabolites associated with neurotransmission pathways analyzed in the targeted approach by Markin et al. [34].

Neurotransmitter System and Metabolite Pathways	Metabolites
GABA/Glu innervation	GABA, Glu, Gln
Serotonergic system/SER pathway	Trp, 5-hydroytryptophan, SER, 5-hydroxyindole acetic acid, tryptamine
Dopaminergic/adrenergic system	Phe, Tyr, L-DOPA, DA, NE, normetanephrine, epinephrine, metanephrine
Asp innervation system	Asp, Asn
Cholinergic system	Acetylcholine, choline
Kynurenine pathway	Kynurenine, kynurenic acid, anthranilic acid, xanthurenic acid, quinolinic acid, picolinic acid
Microbial Trp catabolism	Indole-3-carboxaldehyde, indole-3-acetic acid, indole-3-butyric acid, indole-3-lactic acid, indole-3-acrylic acid, indole-3-propionic acid
Others	Cortisol, citrulline, biopterin, neopterin

DA: Dopamine; GABA: γ-Aminobutyric acid; NE: Norepinephrine; SER: Serotonin.

## Data Availability

Not applicable.

## References

[1] Zanda M.T., Fattore L., Watson R.R., Zibadi S. (2017). Chapter 29—Novel Psychoactive Substances: A New Behavioral and Mental Health Threat. Addictive Substances and Neurological Disease.

[2] Coppola M., Mondola R., Oliva F., Picci R.L., Ascheri D., Trivelli F., Preedy V.R. (2016). Chapter 63—Treating the Phenomenon of New Psychoactive Substances: Synthetic Cannabinoids and Synthetic Cathinones. Neuropathology of Drug Addictions and Substance Misuse.

[3] Zawilska J.B., Taba P., Lees A., Sikk K. (2015). Chapter Thirteen—“Legal Highs”—An Emerging Epidemic of Novel Psychoactive Substances. International Review of Neurobiology.

[4] EMCDDA New Psychoactive Substances—The Current Situation in Europe (European Drug Report 2024). https://www.euda.europa.eu/publications/european-drug-report/2024/new-psychoactive-substances_en.

[5] Majchrzak M., Celiński R., Kuś P., Kowalska T., Sajewicz M. (2018). The newest cathinone derivatives as designer drugs: An analytical and toxicological review. Forensic Toxicol..

[6] Paillet-Loilier M., Cesbron A., Boisselier R., Bourgine J., Debruyne D. (2014). Emerging drugs of abuse: Current perspectives on substituted cathinones. Subst. Abus. Rehabil..

[7] Young R., Glennon R.A. (1993). Cocaine-stimulus generalization to two new designer drugs: Methcathinone and 4-methylaminorex. Pharmacol. Biochem. Behav..

[8] Valente M.J., Guedes de Pinho P., de Lourdes Bastos M., Carvalho F., Carvalho M. (2014). Khat and synthetic cathinones: A review. Arch. Toxicol..

[9] German C.L., Fleckenstein A.E., Hanson G.R. (2014). Bath salts and synthetic cathinones: An emerging designer drug phenomenon. Life Sci..

[10] Gonçalves J.L., Alves V.L., Aguiar J., Teixeira H.M., Câmara J.S. (2019). Synthetic cathinones: An evolving class of new psychoactive substances. Crit. Rev. Toxicol..

[11] Debruyne D., Le Boisselier R. (2015). Emerging drugs of abuse: Current perspectives on synthetic cannabinoids. Subst. Abus. Rehabil..

[12] Papaseit E., Pérez-Mañá C., Pérez-Acevedo A.P., Hladun O., Torres-Moreno M.C., Muga R., Torrens M., Farré M. (2018). Cannabinoids: From pot to lab. Int. J. Med. Sci..

[13] Alves V.L., Gonçalves J.L., Aguiar J., Teixeira H.M., Câmara J.S. (2020). The synthetic cannabinoids phenomenon: From structure to toxicological properties. A review. Crit. Rev. Toxicol..

[14] EMCDDA Synthetic Cannabinoids in Europe (Perspectives on Drugs). https://www.emcdda.europa.eu/topics/pods/synthetic-cannabinoids_en.

[15] de Oliveira M.C., Vides M.C., Lassi D.L.S., Torales J., Ventriglio A., Bombana H.S., Leyton V., Périco C.A., Negrão A.B., Malbergier A. (2023). Toxicity of Synthetic Cannabinoids in K2/Spice: A Systematic Review. Brain Sci..

[16] Almeida A.S., Silva B., Pinho P.G., Remião F., Fernandes C. (2022). Synthetic Cathinones: Recent Developments, Enantioselectivity Studies and Enantioseparation Methods. Molecules.

[17] Santos I.C., Maia D., Dinis-Oliveira R.J., Barbosa D.J. (2024). New Psychoactive Substances: Health and Legal Challenges. Psychoactives.

[18] Araújo A.M., Carvalho F., Guedes de Pinho P., Carvalho M. (2021). Toxicometabolomics: Small Molecules to Answer Big Toxicological Questions. Metabolites.

[19] Bouhifd M., Hartung T., Hogberg H.T., Kleensang A., Zhao L. (2013). Review: Toxicometabolomics. J. Appl. Toxicol..

[20] Ramirez T., Daneshian M., Kamp H., Bois F.Y., Clench M.R., Coen M., Donley B., Fischer S.M., Ekman D.R., Fabian E. (2013). Metabolomics in toxicology and preclinical research. Altex.

[21] Zaitsu K., Hayashi Y., Kusano M., Tsuchihashi H., Ishii A. (2016). Application of metabolomics to toxicology of drugs of abuse: A mini review of metabolomics approach to acute and chronic toxicity studies. Drug. Metab. Pharmacokinet..

[22] Araújo A.M., Carvalho M., Costa V.M., Duarte J.A., Dinis-Oliveira R.J., Bastos M.L., Guedes de Pinho P., Carvalho F. (2021). In vivo toxicometabolomics reveals multi-organ and urine metabolic changes in mice upon acute exposure to human-relevant doses of 3,4-methylenedioxypyrovalerone (MDPV). Arch. Toxicol..

[23] Page M.J., McKenzie J.E., Bossuyt P.M., Boutron I., Hoffmann T.C., Mulrow C.D., Shamseer L., Tetzlaff J.M., Akl E.A., Brennan S.E. (2021). The PRISMA 2020 statement: An updated guideline for reporting systematic reviews. BMJ.

[24] Manier S.K., Keller A., Schäper J., Meyer M.R. (2019). Untargeted metabolomics by high resolution mass spectrometry coupled to normal and reversed phase liquid chromatography as a tool to study the in vitro biotransformation of new psychoactive substances. Sci. Rep..

[25] Kim J.H., Kong T.Y., Moon J.Y., Choi K.H., Cho Y.Y., Kang H.C., Lee J.Y., Lee H.S. (2018). Targeted and non-targeted metabolite identification of MAM-2201 in human, mouse, and rat hepatocytes. Drug Test. Anal..

[26] Wang Y., Yang Y., Zhan Y., Yin J., Zhou X., Xu C., Gao F., Liu J., Wu C., Liu S. (2023). Pharmacokinetics and metabolomics of the new psychoactive substance 4-chloroethylcathinone. Arab. J. Chem..

[27] Hemmer S., Wagmann L., Pulver B., Westphal F., Meyer M.R. (2022). In Vitro and In Vivo Toxicometabolomics of the Synthetic Cathinone PCYP Studied by Means of LC-HRMS/MS. Metabolites.

[28] Araújo A.M., Bastos M.D.L., Carvalho F., Guedes de Pinho P., Carvalho M. (2020). Effect of temperature on 3,4-Methylenedioxypyrovalerone (MDPV)-induced metabolome disruption in primary mouse hepatic cells. Toxicology.

[29] Steuer A.E., Kaelin D., Boxler M.I., Eisenbeiss L., Holze F., Vizeli P., Czerwinska J., Dargan P.I., Abbate V., Liechti M.E. (2020). Comparative Untargeted Metabolomics Analysis of the Psychostimulants 3,4-Methylenedioxy-Methamphetamine (MDMA), Amphetamine, and the Novel Psychoactive Substance Mephedrone after Controlled Drug Administration to Humans. Metabolites.

[30] Olesti E., De Toma I., Ramaekers J.G., Brunt T.M., Carbó M.L., Fernández-Avilés C., Robledo P., Farré M., Dierssen M., Pozo Ó.J. (2019). Metabolomics predicts the pharmacological profile of new psychoactive substances. J. Psychopharmacol..

[31] Manier S.K., Wagmann L., Flockerzi V., Meyer M.R. (2020). Toxicometabolomics of the new psychoactive substances α-PBP and α-PEP studied in HepaRG cell incubates by means of untargeted metabolomics revealed unexpected amino acid adducts. Arch. Toxicol..

[32] Araújo A.M., Carvalho M., Bastos M.L., Carvalho F., de Pinho P.G. (2019). Metabolic signature of methylone in primary mouse hepatocytes, at subtoxic concentrations. Arch. Toxicol..

[33] Shestakova K.M., Mesonzhnik N.V., Markin P.A., Moskaleva N.E., Nedorubov A.A., Brito A., Appolonova E.G., Kuznetsov R.M., Bochkareva N.L., Kukharenko A. (2021). Pharmacokinetic Properties of the Novel Synthetic Cannabinoid 5F-APINAC and Its Influence on Metabolites Associated with Neurotransmission in Rabbit Plasma. Pharmaceuticals.

[34] Markin P.A., Brito A., Moskaleva N.E., Tagliaro F., La Frano M.R., Savitskii M.V., Appolonova S.A. (2021). Short- and long-term exposures of the synthetic cannabinoid 5F-APINAC induce metabolomic alterations associated with neurotransmitter systems and embryotoxicity confirmed by teratogenicity in zebrafish. Comp. Biochem. Physiol. C Toxicol. Pharmacol..

[35] Li R.S., Fukumori R., Takeda T., Song Y., Morimoto S., Kikura-Hanajiri R., Yamaguchi T., Watanabe K., Aritake K., Tanaka Y. (2019). Elevation of endocannabinoids in the brain by synthetic cannabinoid JWH-018: Mechanism and effect on learning and memory. Sci. Rep..

[36] Zaitsu K., Hayashi Y., Suzuki K., Nakayama H., Hattori N., Takahara R., Kusano M., Tsuchihashi H., Ishii A. (2015). Metabolome disruption of the rat cerebrum induced by the acute toxic effects of the synthetic cannabinoid MAM-2201. Life Sci..

[37] Nagana Gowda G.A., Raftery D. (2021). NMR-Based Metabolomics. Adv. Exp. Med. Biol..

[38] Emwas A.H., Roy R., McKay R.T., Tenori L., Saccenti E., Gowda G.A.N., Raftery D., Alahmari F., Jaremko L., Jaremko M. (2019). NMR Spectroscopy for Metabolomics Research. Metabolites.

[39] Wishart D.S., Cheng L.L., Copié V., Edison A.S., Eghbalnia H.R., Hoch J.C., Gouveia G.J., Pathmasiri W., Powers R., Schock T.B. (2022). NMR and Metabolomics-A Roadmap for the Future. Metabolites.

[40] Crook A.A., Powers R. (2020). Quantitative NMR-Based Biomedical Metabolomics: Current Status and Applications. Molecules.

[41] Zeki Ö.C., Eylem C.C., Reçber T., Kır S., Nemutlu E. (2020). Integration of GC-MS and LC-MS for untargeted metabolomics profiling. J. Pharm. Biomed. Anal..

[42] Zhang X.W., Li Q.H., Xu Z.D., Dou J.J. (2020). Mass spectrometry-based metabolomics in health and medical science: A systematic review. RSC Adv..

[43] Bujak R., Struck-Lewicka W., Markuszewski M.J., Kaliszan R. (2015). Metabolomics for laboratory diagnostics. J. Pharm. Biomed. Anal..

[44] Szeremeta M., Pietrowska K., Niemcunowicz-Janica A., Kretowski A., Ciborowski M. (2021). Applications of Metabolomics in Forensic Toxicology and Forensic Medicine. Int. J. Mol. Sci..

[45] Johnson C.H., Ivanisevic J., Siuzdak G. (2016). Metabolomics: Beyond biomarkers and towards mechanisms. Nat. Rev. Mol. Cell Biol..

[46] Baumann M.H., Walters H.M., Niello M., Sitte H.H. (2018). Neuropharmacology of Synthetic Cathinones. Handb. Exp. Pharmacol..

[47] Saeidnia S., Manayi A., Abdollahi M. (2015). From in vitro Experiments to in vivo and Clinical Studies; Pros and Cons. Curr. Drug. Discov. Technol..

[48] Zhang A., Sun H., Xu H., Qiu S., Wang X. (2013). Cell metabolomics. Omics.

[49] Hartung T., Daston G. (2009). Are in vitro tests suitable for regulatory use?. Toxicol. Sci..

[50] Valente M.J., Araújo A.M., Silva R., Bastos M.d.L., Carvalho F., Guedes de Pinho P., Carvalho M. (2016). 3,4-Methylenedioxypyrovalerone (MDPV): In vitro mechanisms of hepatotoxicity under normothermic and hyperthermic conditions. Arch. Toxicol..

[51] Luethi D., Liechti M.E., Krähenbühl S. (2017). Mechanisms of hepatocellular toxicity associated with new psychoactive synthetic cathinones. Toxicology.

[52] Chetwynd A.J., Dunn W.B., Rodriguez-Blanco G. (2017). Collection and Preparation of Clinical Samples for Metabolomics. Adv. Exp. Med. Biol..

[53] Smith L., Villaret-Cazadamont J., Claus S.P., Canlet C., Guillou H., Cabaton N.J., Ellero-Simatos S. (2020). Important Considerations for Sample Collection in Metabolomics Studies with a Special Focus on Applications to Liver Functions. Metabolites.

[54] Garibotto G., Sofia A., Saffioti S., Bonanni A., Mannucci I., Verzola D. (2010). Amino acid and protein metabolism in the human kidney and in patients with chronic kidney disease. Clin. Nutr..

[55] Ohno-Shosaku T., Maejima T., Kano M. (2001). Endogenous cannabinoids mediate retrograde signals from depolarized postsynaptic neurons to presynaptic terminals. Neuron.

[56] Barbieri M., Ossato A., Canazza I., Trapella C., Borelli A.C., Beggiato S., Rimondo C., Serpelloni G., Ferraro L., Marti M. (2016). Synthetic cannabinoid JWH-018 and its halogenated derivatives JWH-018-Cl and JWH-018-Br impair Novel Object Recognition in mice: Behavioral, electrophysiological and neurochemical evidence. Neuropharmacology.

[57] Iversen L. (2003). Cannabis and the brain. Brain.

[58] Sigel E., Baur R., Rácz I., Marazzi J., Smart T.G., Zimmer A., Gertsch J. (2011). The major central endocannabinoid directly acts at GABA(A) receptors. Proc. Natl. Acad. Sci. USA.

[59] Altun B., Çok İ. (2020). Psychoactive Bath Salts and Neurotoxicity Risk. Turk. J. Pharma. Sci..

[60] Araújo A.M., Bastos M.L., Fernandes E., Carvalho F., Carvalho M., Guedes de Pinho P. (2018). GC-MS metabolomics reveals disturbed metabolic pathways in primary mouse hepatocytes exposed to subtoxic levels of 3,4-methylenedioxymethamphetamine (MDMA). Arch. Toxicol..

[61] Perrine S.A., Michaels M.S., Ghoddoussi F., Hyde E.M., Tancer M.E., Galloway M.P. (2009). Cardiac effects of MDMA on the metabolic profile determined with 1H-magnetic resonance spectroscopy in the rat. NMR Biomed..

[62] Bloomfield M.A., Ashok A.H., Volkow N.D., Howes O.D. (2016). The effects of Δ(9)-tetrahydrocannabinol on the dopamine system. Nature.

[63] Moranta D., Esteban S., García-Sevilla J.A. (2004). Differential effects of acute cannabinoid drug treatment, mediated by CB1 receptors, on the in vivo activity of tyrosine and tryptophan hydroxylase in the rat brain. Naunyn Schmiedebergs Arch. Pharmacol..

[64] Dando I., Donadelli M., Costanzo C., Dalla Pozza E., D’Alessandro A., Zolla L., Palmieri M. (2013). Cannabinoids inhibit energetic metabolism and induce AMPK-dependent autophagy in pancreatic cancer cells. Cell Death Dis..

